# Idiopathic Pulmonary Hemosiderosis in a Child with Recurrent Macrophage Activation Syndrome Secondary to Systemic Juvenile Idiopathic Arthritis

**DOI:** 10.1155/2017/5693501

**Published:** 2017-01-30

**Authors:** Kenan Barut, Sezgin Sahin, Amra Adrovic, Velat Sen, Ozgur Kasapcopur

**Affiliations:** ^1^Department of Pediatric Rheumatology, Cerrahpasa Medical School, Istanbul University, Istanbul, Turkey; ^2^Department of Pediatric Chest Disease, Medical Faculty, Dicle University, Diyarbakir, Turkey

## Abstract

Macrophage activation syndrome, a severe complication of systemic juvenile idiopathic arthritis and other inflammatory diseases, represents one of the most important rheumatological emergencies. Delayed diagnosis could lead to life-threatening complications. Pulmonary hemosiderosis has been classically characterized by a triad of anemia, hemoptysis, and lung infiltrates on chest radiogram. Although the majority of patients of pulmonary hemosiderosis are considered idiopathic, secondary hemosiderosis associated with known diseases could be seen. In this case report, we aimed to present gradually increased pulmonary manifestations due to pulmonary hemosiderosis with recurrent macrophage activation syndrome attacks in a child with systemic juvenile idiopathic arthritis.

## 1. Background

Systemic juvenile idiopathic arthritis (SJIA) is a rare disease characterized by prolonged and intermittent fever, arthritis, and evanescent rash. Other features in a minority of patients are hepatosplenomegaly, lymphadenopathy, and serositis [1]. Macrophage activation syndrome (MAS) is the most important and destructive complication of the disease. MAS represents an acquired hemophagocytosis secondary to rheumatological, infectious, oncological, and immune deficiency diseases [2]. The main clinical features of this lethal disease are transition of intermittent fever into a continued type, hepatosplenomegaly, enlarged lymph nodes, neurological findings, purpura, and mucosal hemorrhages. Laboratory features are gradually decreasing WBC/platelet counts and fibrinogen levels associated with decreased ESR. Usually liver enzymes, triglycerides, and ferritin levels are also elevated [3].

Idiopathic pulmonary hemosiderosis (IPH) is a rare condition that could be seen from the neonatal period until the adulthood; initial symptoms emerge in the first decade of life in the 80% of patients, especially in the first 7 years. In the literature, the most commonly reported cases are children with the estimated incidence of IPH of 0.24–1.23 per million and mortality rate of 50% [4]. Recurrent intra-alveolar hemorrhage leading to accumulation of hemosiderin and interstitial fibrosis is the possible pathophysiological mechanism for IPH. The aetiology of IPH remains unknown [4, 5]. There are a few reports of IPH cases secondary to the autoimmune diseases such as celiac disease and vasculitis [6, 7]. Up to date two cases of IPH with juvenile idiopathic arthritis have been reported [8, 9]. Clinical presentation of the disease is a triad of hemoptysis, dyspnea, and varying degrees of anemia. Chest radiography reveals a widespread pulmonary edema and/or patchy opacities in lung [10].

In this case report, we present a case of pulmonary hemosiderosis with recurrent macrophage activation syndrome secondary to systemic juvenile idiopathic arthritis which has not been reported previously in the literature.

## 2. Case Presentation

A 13-month-old previously healthy infant was admitted to hospital because of high fever and rash. The fever showed an intermittent course, lasting for 2 weeks. Findings on physical examination at admission were poor general condition, marked hepatosplenomegaly (enlarged liver 4 cm, spleen 3 cm), bilateral wrist and ankle arthritis, and a maculopapular, pink coloured rash that is more prominent especially during high fever.

Complete blood count revealed marked leukocytosis (WBC: 35.000/mm^3^) and anemia (Hgb 7.7 g/dL) with normal platelet level (PLT: 356.000/mm^3^). Ferritin level was increased to 24.282 ng/mL. Elevated erythrocyte sedimentation rate (ESR) (39 mm/h) and C-reactive protein (CRP) level (6.8 mg/dL) (nephelometry; normal < 0.5 mg/dL) were present. While levels of aspartate aminotransferase (AST: 68 IU/L) and lactate dehydrogenase (LDH: 1133 IU/L) were increased, hypofibrinogenemia (fibrinogen: 35 ng/mL) and hypertriglyceridemia (triglyceride: 258 mg/dL) were remarkable in laboratory studies. Bone marrow aspiration was performed; no hemophagocytic cells were found.

In order to exclude the bacterial and viral infectious diseases, blood and bone marrow cultures along with viral serological tests were performed and they were found to be negative. We have considered malignancy and metabolic storage diseases in the differential diagnosis of this patient and performed bone marrow biopsy. Neither storage cells nor the malignant cells were found. The history of the disease and clinical presentation were highly suggestive for diagnosis of SJIA and MAS secondary to SJIA.

Pulse steroid (a dose of 30 mg/kg methylprednisolone intravenously for 3 days), methotrexate (MTX), and cyclosporine-A treatment were administered.

**Figure 1:**
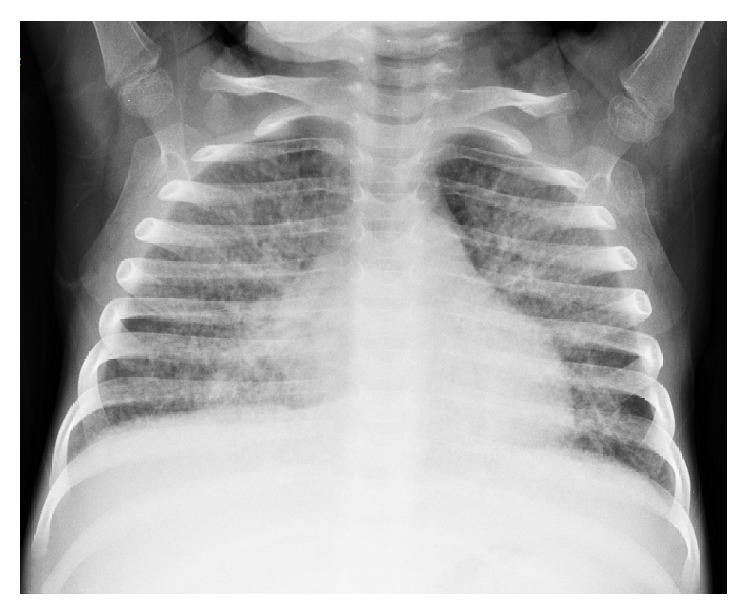
Bilateral lung interstitial infiltration in chest radiography.

A diagnosis of MAS secondary to SJIA was confirmed for about five times during the one-year follow-up. At the time of the last MAS attack, a ferritin level was found to be as high as 120.990 ng/mL. Due to recurrent MAS attacks, a genetic investigation for familial hemophagocytic lymphohistiocytosis was performed; however no mutation was found. During the last hospitalization of the patient when she was 2.5 years old, respiratory distress was noticed in physical examination. Diffuse infiltrations on chest radiography (Figure 1) with iron deficiency anemia were reasons to consider pulmonary hemosiderosis in the differential diagnosis of this condition.

**Figure 2:**
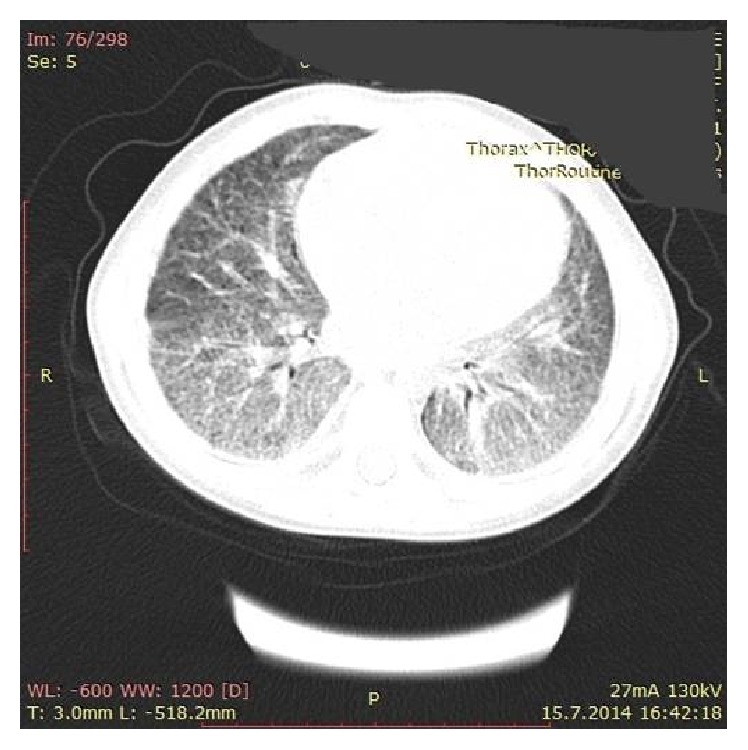
Bilateral diffuse fibrous changes and reticulonodular opacities in chest tomogram.

Thoracic computerized tomography revealed diffuse fibrotic changes and reticulonodular opacities in the lung (Figure 2). Histochemical investigation of bronchoalveolar lavage fluid by the ferrous stain showed iron-bearing macrophages above 20%. Thereby, these features suggested a diagnosis of IPH with recurrent MAS attacks.

Again pulse methylprednisolone therapy was given intravenously for 3 days to control the last MAS attack. In addition, prednisolone (1 mg/kg/day orally), MTX (15 mg/m^2^/week orally), cyclosporine-A (5 mg/kg/day orally), and anti-IL1 (Anakinra 5 mg/kg/day subcutaneously) were used in maintenance therapy. During the further clinical course of the disease, dyspnea became more prominent so the patient was admitted to the intensive care unit. At the time of discharge from the hospital, patient remained dependent on oxygen therapy. With the mentioned therapy general condition of the patient has been improved and ultimately ferritin level was decreased to 773 ng/mL after treatment. She has never experienced such a MAS attack; however oxygen dependence has persisted. While the patient was under the maintenance therapy, she died of respiratory insufficiency when she was 3.5 years old.

## 3. Discussion

Pulmonary hemosiderosis is being divided into two main group: idiopathic (primary) and secondary PH. IPH is considered to be more common than secondary. Collagen vascular diseases, coagulation disorders, and cardiological diseases, especially mitral stenosis, were described as the most common causes of secondary PH. Additionally, repeated blood transfusions in patients with thalassemia represent an important reason for secondary PH [4]. In the case of MAS, the most important and the most destructive complication of SJIA, ferritin could reach a very high level. Recently conducted multicentric study among SJIA secondary MAS cases showed a mean initial ferritin level to be as high as 8,325 ng/mL (2,048–22,977 ng/mL) [3]. An investigation of MAS patients secondary to SJIA that were being followed up at our department during last year showed a mean ferritin level of 23957 ± 15525 ng/mL (minimal: 3000 ng/mL, maximal: 46.130 ng/mL). None of those patients developed pulmonary hemosiderosis [1]. The serum ferritin levels could be elevated or it could be in normal limits in IPH. Increased serum ferritin levels do not indicate iron storage in alveoli [5]. We can speculate that pulmonary hemosiderosis in our patients is related to extremely high level of serum ferritin.

Kiper et al. [10] reported multiple blood transfusions in 10 of 23 pulmonary hemosiderosis patients. The development of PH in these patients is attributed to high ferritin levels secondary to blood transfusions. This finding could explain the association of increased ferritin levels with PH in our patient also.

The role of autoimmunity in the pathophysiological process of IPH is not totally clear [5]. Besides, coexistence of IPH with various autoimmune diseases has been reported. In the current literature, there are two case reports of PH associated with coeliac disease [6, 7], two cases with juvenile idiopathic arthritis (seronegative polyarticular and suspicious oligoarticular JIA) [8, 9], and one case with lupus [11]. To our knowledge, IPH with systemic JIA has not been reported in literature. Our patient was classified as SJIA subset of JIA due to presence of systemic manifestations such as fever, rash, and organomegaly. Although the previously reported cases [8, 9] and our patient were not classified as the same JIA subtype, the pathophysiological nature of both diseases suggests autoimmunity in etiology of IPH.

The diagnosis of PH is confirmed by presence of hemosiderin-bearing macrophages in bronchoalveolar lavage fluids or at lung biopsy materials, without underlying vasculitis, nonspecific granulomatous inflammation, or deposits of immunoglobulin [5].

In a multicenter study by Kimura et al. [12], lung involvement was detected in 25 SJIA patients. Detailed investigation of these patients revealed that 64% (*n* = 16) of them had pulmonary arterial hypertension (PAH), 28% (*n* = 7) had interstitial lung disease (IPL), and 20% (*n* = 5) had alveolar proteinosis (AP). Severe iron deficiency anemia and typical HRCT findings in our patient have led us to suspect IPH other than these lung involvement patterns. Percentage of hemosiderin-laden macrophages in BAL as above 20% supported our preliminary diagnosis. Serial echocardiographic assessments in a 2-year period did not show any sign of PAH finding. HRCT findings and milk-like fluid in BAL material that are typical for AP diagnosis have not been detected in our patient [13]. Although the clinical manifestations and the presence of hemosiderin-laden macrophages in bronchoalveolar lavage are suggestive for PH diagnosis, it is often nondiagnostic in pediatric patients. Lung biopsy is usually indicated to rule out collagen tissue diseases and vasculitis. However, rapid deterioration of respiratory distress did not give us an opportunity to perform the biopsy procedure in our patient.

Classification criteria for SJIA associated MAS have been validated recently based on the following features: A ferritin level more than 684 ng/mL plus presence of two of the following: platelet count ≤ 181,000/mm^3^, fibrinogen ≤ 360 mg/dL, aspartate aminotransferase > 48 IU/L, and triglyceride > 156 mg/dL [14]. Both clinical and laboratory manifestations of our patient were in accordance with these criteria.

Likewise, we have managed the MAS attack by standard treatment protocol. However, the same medications were insufficient in controlling the deterioration of respiratory distress as respiratory insufficiency became more apparent by the time.

Iron deficiency anemia is frequently seen in IPH patients due to alveolar hemorrhagia [5].

Hypochromic microcytic anemia in our patient was attributed to iron deficiency as a consequence of alveolar hemorrhagia and also to the chronic inflammation related decrease in production of haemoglobin. Apparent hemoptysis has never been seen in our case.

Chronic cough, dyspnea, hemoptysis, and fatigue are initial clinical manifestations of IPH and asymptomatic anemia may be seen, occasionally [5]. Diffuse alveolar infiltrations on chest X-ray and ground-glass appearance on chest CT are common radiological findings in the early phase of the disease. Reticulonodular image could be seen after resorption of alveolar hemorrhage in remission phase [5]. In our patient, bilateral alveolar infiltrations in basal segments of the lung followed by reticulonodular pattern were registered on the chest CT.

Based on the clinical suspicion, bronchoalveolar lavage was performed and it revealed hemosiderin-laden macrophages.

To the best of our knowledge, this is the first case of IPH with MAS attacks that has been reported in medical literature. Since IPH could be a possible complication of MAS, it should be considered in relapsing MAS patients, particularly in those with respiratory symptoms.

In conclusion, thrombocytopenia and gradually increasing ferritin levels despite the treatment may indicate development of MAS in a SJIA patient. Rheumatologists dealing with chronic diseases should be aware of extraordinary manifestations of these rare diseases, as in our case. For instance, dyspnea, pulmonary infiltrations on chest radiography, and varying degrees of anemia in a SJIA associated recurrent MAS patient should alert the physician for the possibility of IPH. There could be speculations regarding the development of IPH in this particular case; whether it is a consequence of hyperferritinemia of recurrent MAS attacks or this case is an example of a novel autoinflammatory disorder. Reporting bizarre manifestations of rheumatological diseases, especially in suspected autoinflammatory disorders, could lead to definition of new diseases in the era of autoinflammation.

## Abbreviations

IPH: Idiopathic pulmonary hemosiderosis
SJIA: Systemic juvenile idiopathic arthritis
BAL: Bronchoalveolar lavage
MAS: Macrophage activation syndrome
MTX: Methotrexate
PAH: Pulmonary arterial hypertension
AP: Alveolar proteinosis
IPL: Interstitial lung disease.
